# Induction of Suicidal Erythrocyte Death by Nelfinavir

**DOI:** 10.3390/toxins7051616

**Published:** 2015-05-08

**Authors:** Rosi Bissinger, Sabrina Waibel, Florian Lang

**Affiliations:** Department of Physiology, University of Tuebingen, Gmelinstr. 5, 72076 Tuebingen, Germany; E-Mails: ro.bissinger@gmx.de (R.B.); Sabi-W@gmx.de (S.W.)

**Keywords:** phosphatidylserine, calcium, cell volume, ROS, oxidative stress, eryptosis, malaria

## Abstract

The HIV protease inhibitor, nelfinavir, primarily used for the treatment of HIV infections, has later been shown to be effective in various infectious diseases including malaria. Nelfinavir may trigger mitochondria-independent cell death. Erythrocytes may undergo eryptosis, a mitochondria-independent suicidal cell death characterized by cell shrinkage and phosphatidylserine translocation to the erythrocyte surface. Triggers of eryptosis include oxidative stress and increase of cytosolic Ca^2+^**-**activity ([Ca^2+^]_i_). During malaria, accelerated death of infected erythrocytes may decrease parasitemia and thus favorably influence the clinical course of the disease. In the present study, phosphatidylserine abundance at the cell surface was estimated from annexin V binding, cell volume from forward scatter, reactive oxidant species (ROS) from 2',7'-dichlorodihydrofluorescein diacetate (DCFDA) fluorescence, and [Ca^2+^]_i_ from Fluo3-fluorescence. A 48 h treatment of human erythrocytes with nelfinavir significantly increased the percentage of annexin-V-binding cells (≥5µg/mL), significantly decreased forward scatter (≥2.5µg/mL), significantly increased ROS abundance (10 µg/mL), and significantly increased [Ca^2+^]_i_ (≥5 µg/mL). The up-regulation of annexin-V-binding following nelfinavir treatment was significantly blunted, but not abolished by either addition of the antioxidant N-acetylcysteine (1 mM) or removal of extracellular Ca^2+^. In conclusion, exposure of erythrocytes to nelfinavir induces oxidative stress and Ca^2+^ entry, thus leading to suicidal erythrocyte death characterized by erythrocyte shrinkage and erythrocyte membrane scrambling.

## 1. Introduction

Nelfinavir, a specific HIV protease inhibitor, has originally been developed for the treatment of HIV infections and subsequently been shown to be effective in further infectious diseases including SARS, tuberculosis, and malaria [1,2,3,4,5]. Beyond that, nelfinavir may trigger death of tumor cells and thus counteracts malignancy [2,3,6,7]. Nelfinavir is in part effective by triggering of mitochondria-independent apoptosis [2,3].

In analogy to apoptosis of nucleated cells, erythrocytes could enter eryptosis, a suicidal death characterized by cell shrinkage [8] and translocation of phosphatidylserine from the cell interior to the erythrocyte surface [9]. Cellular mechanisms involved in the stimulation of eryptosis include oxidative stress [9], increased cytosolic Ca^2+^ activity ([Ca^2+^]_i_), ceramide [10], energy depletion [9], and activated caspases [9,11,12]. Moreover, eryptosis may be stimulated by casein kinase 1α, Janus-activated kinase JAK3, protein kinase C, p38 kinase, and PAK2 kinase [9]. Eryptosis is inhibited by AMP activated kinase AMPK, cGMP-dependent protein kinase, sorafenib and sunitinib sensitive kinases [9]. Eryptosis is distinct from programmed erythrocyte necrosis [13], which is triggered by pore-forming bacterial toxins.

Eryptosis is stimulated by diverse xenobiotics [9,14,15,16,17,18,19,20,21,22,23,24,25,26,27,28,29,30,31,32,33,34,35,36,37,38,39,40,41,42,43,44,45,46,47,48,49,50]. Eryptosis is further triggered during malaria and accelerated eryptosis favourably influences the clinical course of the disease [51].

The present study explored, whether and how nelfinavir stimulates eryptosis. To this end, erythrocytes from healthy volunteers were exposed to nelfinavir and phosphatidylserine abundance at the erythrocyte surface, cell volume, abundance of reactive oxidant species and [Ca^2+^]_i_ determined utilizing flow cytometry.

## 2. Results

**Figure 1:**
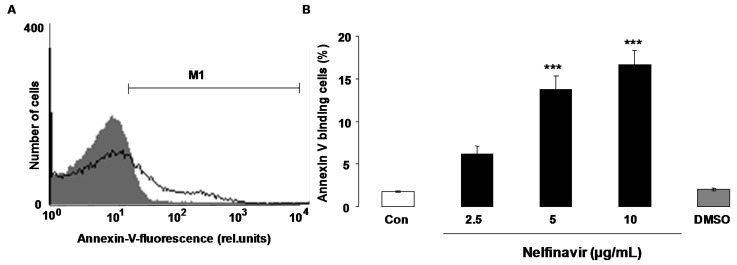
Effect of nelfinavir on phosphatidylserine exposure. (**A**) Original histogram of annexin-V-binding of erythrocytes following exposure for 48 h to Ringer solution without (grey area) and with (black line) presence of 10 µg/mL nelfinavir. M1 indicates the annexin-V-fluoresence defining the percentage of annexin-V-binding erythrocytes; (**B**) Arithmetic means ± SEM of erythrocyte annexin-V-binding (*n* = 15) following incubation for 48 h to Ringer solution without (white bar) or with (black bars) presence of nelfinavir (2.5–10 µg/mL). For comparison, the effect of the solvent DMSO (1 µL/mL Ringer) is shown (grey bar). *** (*p* < 0.001) indicates significant difference from the absence of nelfinavir (ANOVA).

The present study explored whether nelfinavir is capable to trigger eryptosis, the suicidal erythrocyte death. Hallmarks of eryptosis are cell shrinkage and phospholipid scrambling of the cell membrane with phosphatidylserine translocation to the cell surface. In order to quantify phospholipid scrambling of the cell membrane, phosphatidylserine abundance at the cell surface was quantified by determination of phosphatidylserine-binding FITC-labelled annexin-V in flow cytometry. As shown in Figure 1, a 48 h exposure to nelfinavir increased the percentage of annexin-V-binding erythrocytes, an effect reaching statistical significance at 5 μg/mL nelfinavir concentration. Hemoglobin concentration in the supernatant was determined in order to estimate the effect of nelfinavir on hemolysis. According to hemoglobin concentration in the supernatant, a 48 h incubation with 0, 2.5, 5 and 10 μg/mL Nelfinavir resulted in hemolysis of 1.9% ± 0.3%, 3.5% ± 0.3%. 4.0% ± 0.2% and 7.0% ± 1.0% (*n* = 5), respectively.

**Figure 2:**
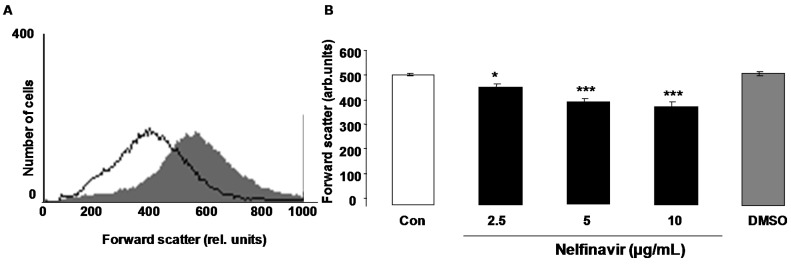
Effect of nelfinavir on erythrocyte forward scatter. (**A**) Original histogram of forward scatter of erythrocytes following exposure for 48 h to Ringer solution without (grey area) and with (black line) presence of 10 µg/mL nelfinavir; (**B**) Arithmetic means ± SEM (*n* = 15) of the erythrocyte forward scatter (FSC) following incubation for 48 h to Ringer solution without (white bar) or with (black bars) nelfinavir (2.5–10 µg/mL). For comparison, the effect of the solvent DMSO (1 µL/mL Ringer) is shown (grey bar). * (*p* < 0.05), *** (*p* < 0.001) indicate significant difference from the absence of nelfinavir (ANOVA).

Erythrocyte cell volume was estimated from forward scatter in flow cytometry. As illustrated in Figure 2, a 48 h nelfinavir treatment was followed by a decrease of erythrocyte forward scatter, an effect reaching statistical significance at 2.5 µg/mL nelfinavir concentration.

Nelfinavir treatment thus triggered phospholipid scrambling of the erythrocyte membrane and cell shrinkage, the two hallmarks of eryptosis. Additional experiments were performed to shed light on the cellular mechanisms underlying the triggering of eryptosis.

**Figure 3:**
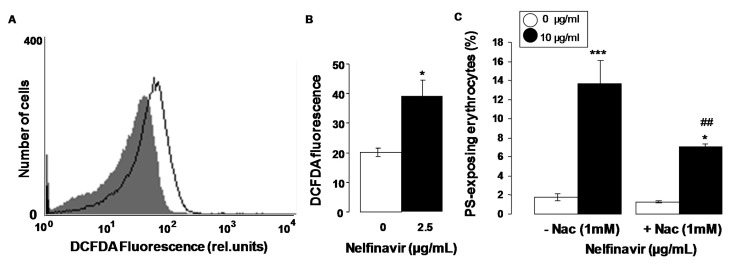
Effect of nelfinavir on reactive oxygen species. (**A**) Original histogram of 2',7'-dichlorodihydrofluorescein diacetate (DCFDA) fluorescence in erythrocytes following exposure for 48 h to Ringer solution without (grey shadow) and with (black line) presence of 10 µg/mL nelfinavir; (**B**) Arithmetic means ± SEM (*n* = 5) of the erythrocyte DCFDA fluorescence following incubation for 48 h to Ringer solution without (white bar) or with (black bar) presence of 10 µg/mL nelfinavir. * (*p*<0.05) indicates significant difference from the absence of nelfinavir (*t* test); (**C**) Arithmetic means ± SEM (*n* = 6) of annexin-V-binding of erythrocytes after a 48 h treatment with Ringer solution without (white bars) or with (black bars) 10 µg/mL nelfinavir in the absence (left bars, −Nac) and presence (right bars, +Nac) of the antioxidant N-acetylcysteine (1 mM). * (*p* < 0.05), *** (*p* < 0.001) indicate significant difference from the absence of nelfinavir, ## (*p* < 0.01) indicates significant difference from the respective value in the absence of the antioxidant N-acetylcysteine (1 mM).

Mechanisms stimulating eryptosis include oxidative stress. Thus, additional experiments explored, whether nelfinavir influences the formation of reactive oxygen species (ROS). To this end, ROS was quantified utilizing 2',7'-dichlorodihydrofluorescein diacetate (DCFDA). As illustrated in Figure 3A,B, a 48 h exposure to nelfinavir (10 µg/mL) was followed by a significant increase of DCFDA fluorescence. Nelfinavir thus induced oxidative stress. An additional series of experiments explored whether nelfinavir-induced translocation of phosphatidylserine to the cell surface required oxidative stress and could thus be abrogated by the reducing substance N-acetylcysteine. To this end, erythrocytes were incubated for 48 h in the absence or presence of 10 µg/mL nelfinavir, both in the absence or presence of N-acetylcysteine (1 mM). As shown in Figure 3C, addition of N-acetylcysteine (1 mM) significantly blunted the effect of nelfinavir on annexin-V-binding, an observation indicating that oxidative stress contributed to the stimulation of cell membrane scrambling by nelfinavir. However, even in the presence of N-acetylcysteine nelfinavir significantly increased the percentage of annexin-V-binding erythrocytes, indicating that eryptosis was in part due to mechanisms other than oxidative stress.

**Figure 4:**
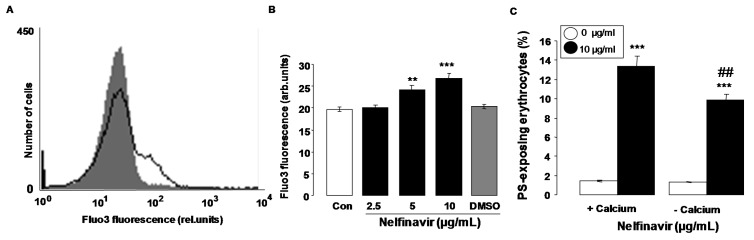
Effect of nelfinavir on erythrocyte Ca^2+^ activity and Ca^2+^ dependence of nelfinavir-induced phosphatidylserine exposure (**A**) Original histogram of Fluo3 fluorescence in erythrocytes following exposure for 48 h to Ringer solution without (grey area) and with (black line) presence of nelfinavir (10 µg/mL); (**B**) Arithmetic means ± SEM (*n* = 15) of the Fluo3 fluorescence (arbitrary units) in erythrocytes exposed for 48 h to Ringer solution without (white bar) or with (black bars) nelfinavir (2.5–10 µg/mL). For comparison, the effect of the solvent DMSO (1 µL/mL Ringer) is shown (grey bar). ** (*p* < 0.01), *** (*p* < 0.001) indicate significant difference from the absence of nelfinavir (ANOVA); (**C**) Arithmetic means ± SEM (*n* = 7) of annexin-V-binding of erythrocytes after a 48 h treatment with Ringer solution without (white bars) or with (black bars) 10 µg/mL nelfinavir in the presence (left bars, +Calcium) and absence (right bars, −Calcium) of Ca^2+^. *** (*p* < 0.001) indicates significant difference from the absence of nelfinavir, ## (*p* < 0.01) indicates significant difference from the respective value in the presence of Ca^2+^.

Oxidative stress is known to activate Ca^2+^ permeable cation channels with subsequent Ca^2+^ entry. Additional experiments thus explored whether nelfinavir influences cytosolic Ca^2+^ activity ([Ca^2+^]_i_). [Ca^2+^]_i_ was quantified utilizing Fluo3 fluorescence. As shown in Figure 4A,B, a 48 h exposure to nelfinavir (2.5–10 µg/mL) increased the Fluo3 fluorescence, an effect reaching statistical significance at 5 µg/mL nelfinavir concentration. An additional series of experiments explored whether nelfinavir-induced translocation of phosphatidylserine to the cell surface required entry of extracellular Ca^2+^. To this end, erythrocytes were incubated for 48 h in the absence or presence of 10 µg/mL nelfinavir, both in the presence or nominal absence of extracellular Ca^2+^. As shown in Figure 4C, removal of extracellular Ca^2+^ significantly blunted the effect of nelfinavir on annexin-V-binding, an observation pointing to a role of Ca^2+^ entry from extracellular space in the stimulation of cell membrane scrambling by nelfinavir. However, even in the absence of extracellular Ca^2+^, nelfinavir significantly increased the percentage of annexin-V-binding erythrocytes. Thus, eryptosis was in part triggered by mechanisms other than entry of extracellular Ca^2+^.

## 3. Discussion

The present observations reveal a novel effect of nelfinavir, *i.e*., the triggering of eryptosis, the suicidal erythrocyte death characterized by cell shrinkage and erythrocyte cell membrane scrambling with phosphatidylserine translocation from the cell interior to the erythrocyte surface. The nelfinavir concentration required for stimulation of erythrocyte cell membrane scrambling (5 µg/mL) was similar to plasma concentrations (10 µg/mL) reported *in vivo* [52]. It must be kept in mind, though, that 98% of nelfinavir is bound to plasma proteins [53] and that the free nelfinavir concentration may be accordingly lower [52]. How binding to erythrocytes competes with binding to plasma proteins, is, however, not known.

The nelfinavir induced erythrocyte shrinkage was presumably secondary to increase of cytosolic Ca^2+^ activity ([Ca^2+^]_i_), which leads to cell shrinkage by activation of Ca^2+^ sensitive K^+^ channels, K^+^ exit, cell membrane hyperpolarization, Cl^−^ exit and thus cellular loss of KCl with osmotically obliged water [8].

The nelfinavir induced cell membrane scrambling was in part due to stimulation of Ca^2+^ entry from extracellular space leading to increase of [Ca^2+^]_i_, a powerful stimulator of cell membrane scrambling with phosphatidylserine translocation [8]. Removal of extracellular Ca^2+^ significantly blunted the stimulation of annexin-V-binding following nelfinavir treatment, indicating that Ca^2+^ entry contributed to the stimulation of nelfinavir induced phosphatidylserine translocation. However, even in the absence of extracellular Ca^2+^ nelfinavir significantly enhanced the phosphatidylserine abundance at the cell surface. Thus, the effect of nelfinavir on Ca^2+^ entry contributed to, but did not fully account for the stimulation of phosphatidylserine translocation. Nelfinavir thus triggered cell membrane scrambling in part through mechanisms other than Ca^2+^ entry.

The effect of nelfinavir on [Ca^2+^]_i_ was presumably in part the result of oxidative stress, which activates oxidant sensitive Ca^2+^ permeable erythrocytic cation channels [9]. Beyond that, oxidative stress may trigger eryptosis by further, rather illdefined mechanisms. Nelfinavir has similarly been reported to induce oxidative stress in nucleated cells [2,3,54,55,56,57,58].

Eryptosis serves to clear defective erythrocytes prior to hemolysis with release of hemoglobin, which may undergo glomerular filtration in the kidney and precipitate in the acidic lumen of renal tubules thus occluding affected nephrons [59]. Eryptosis further serves to remove infected erythrocytes during malaria [51]. The malaria pathogen *Plasmodium* imposes oxidative stress on the host erythrocyte leading to activation of several host cell ion channels including Ca^2+^-permeable erythrocyte cation channels [9,60]. The subsequent Ca^2+^ entry triggers cell membrane scrambling, phosphatidylserine translocation, binding to phosphatidylserine receptors at phagocytes, phagocytosis and thus removal of the infected erythrocytes from circulating blood [51]. Eryptotic removal of infected erythrocytes reduces parasitemia and thus favorably influences the clinical course of malaria. Enhanced susceptibility to eryptosis presumably confers protection against a severe course of malaria in several genetic erythrocyte disorders, such as sickle-cell trait, beta-thalassemia-trait, homozygous Hb-C and homozygous G6PD-deficiency [9,61,62,63]. Accelerated eryptosis further contributes to the protective effect against malaria of iron deficiency [64], lead intoxication [64], treatment with chlorpromazine [65] or presence of NO synthase inhibitors [65]. It is tempting to speculate that induction of eryptosis contributes to the antimalarial effect of nelfinavir. As infected erythrocytes are exposed to oxidative stress [51], they are particularly sensitive to triggers of eryptosis and may thus specifically be eleimninated by eryptosis inducing substances. Clearly, additional experimentation is required to confirm or falsify this speculation.

As phosphatidylserine exposing erythrocytes are engulfed by macrophages and thus rapidly cleared from circulating blood, excessive eryptosis may lead to anemia [9]. Moreover, phosphatidylserine exposing erythrocytes may bind to endothelial cells of the vascular wall [66], trigger blood clotting and induce thrombosis [67,68,69]. Phosphatidylserine exposing erythrocytes thus may compromise microcirculation [10,67,70,71,72,73].

## 4. Experimental Section

### 4.1. Erythrocytes, Solutions and Chemicals

Fresh Lithium-Heparin-anticoagulated blood samples were kindly provided by the blood bank of the University of Tübingen. The study is approved by the ethics committee of the University of Tübingen (184/2003 V). The blood was centrifuged at 120 g for 20 min at 23 °C and the platelets and leukocytes-containing supernatant was disposed. Erythrocytes were incubated *in vitro* for 48 h at a hematocrit of 0.4% in Ringer solution containing (in mM) 125 NaCl, 5 KCl, 1 MgSO_4_, 32 *N*-2-hydroxyethylpiperazine-N-2-ethanesulfonic acid (HEPES), 5 glucose, and 1 CaCl_2_; the pH was adjusted to 7.4 and the temperature kept at 37 °C. Where indicated, erythrocytes were exposed to nelfinavir (Sigma Aldrich, Hamburg, Germany), which was dissolved in DMSO (Carl Roth, Karlsruhe, Germany). For comparison, the effect of 1 µL DMSO/mL Ringer was tested.

### 4.2. Annexin-V-Binding and Forward Scatter

After incubation under the respective experimental condition, a 150 µL cell suspension was washed in Ringer solution containing 5 mM CaCl_2_ and then stained with Annexin-V-FITC (1:200 dilution; ImmunoTools, Friesoythe, Germany) in this solution at 37 °C for 20 min under protection from light. In the following, the forward scatter (FSC) of the cells was determined, and annexin-V fluorescence intensity was measured with an excitation wavelength of 488 nm and an emission wavelength of 530 nm on a FACS Calibur (BD, Heidelberg, Germany). In some experiments erythrocytes were preincubated in Ca^2+^ free solution. For determination of annexin-V-binding, addition of Ca^2+^ was required during the 15 min incubation with FITC-annexin V. Immediately thereafter measurements were done so that the exposure to Ca^2+^ was too short to trigger significant phosphatidylserine translocation.

### 4.3. Reactive Oxidant Species (ROS)

Oxidative stress was determined utilizing 2',7'-dichlorodihydrofluorescein diacetate (DCFDA). After incubation, a 150 µL suspension of erythrocytes was washed in Ringer solution and then stained with DCFDA (Sigma, Schnelldorf, Germany) in Ringer solution containing DCFDA at a final concentration of 10 µM. Erythrocytes were incubated at 37 °C for 30 min in the dark and then washed three times in Ringer solution. The DCFDA-loaded erythrocytes were resuspended in 200 µL Ringer solution, and ROS-dependent fluorescence intensity was measured at an excitation wavelength of 488 nm and an emission wavelength of 530 nm on a FACS Calibur (BD).

### 4.4. Intracellular Ca^2+^

After incubation, a 150 µL cell suspension was washed in Ringer solution and then loaded with Fluo-3/AM (Biotium, Hayward, CA, USA) in Ringer solution containing 5 mM CaCl_2_ and 5 µM Fluo-3/AM. The cells were incubated at 37 °C for 30 min and washed twice in Ringer solution containing 5 mM CaCl_2_. The Fluo-3/AM-loaded erythrocytes were resuspended in 200 µL Ringer. Then, Ca^2+^-dependent fluorescence intensity was measured with an excitation wavelength of 488 nm and an emission wavelength of 530 nm on a FACS Calibur.

### 4.5. Hemolysis

For the determination of hemolysis, the samples were centrifuged (3 min at 1600 rpm, room temperature) after incubation under the respective experimental conditions and the supernatants were harvested. As a measure of hemolysis, the hemoglobin (Hb) concentration of the supernatant was determined photometrically at 405 nm. The absorption of the supernatant of erythrocytes lysed in distilled water was defined as 100% hemolysis. Hemolysis is expressed in % of total hemolysis.

### 4.6. Statistics

Data are expressed as arithmetic means ± SEM. As indicated in the figure legends, statistical analysis was made using ANOVA with Tukey’s test as post-test and *t* test as appropriate. n denotes the number of different erythrocyte specimens studied. Since different erythrocyte specimens used in distinct experiments are differently susceptible to triggers of eryptosis, the same erythrocyte specimens have been used for control and experimental conditions.

## 5. Conclusions

Nelfinavir stimulates eryptosis, the suicidal erythrocyte death characterized by erythrocyte cell membrane scrambling and cell shrinkage. The effect is paralleled by, and at least partially due to, oxidative stress and increase of cytosolic Ca^2+^ activity.
